# "The Hospital" Nursing Mirror

**Published:** 1897-12-25

**Authors:** 


"The Hospital" Nursing Mirror.

Being the Nursing Section of "The Hospital."

[Contributions for this Section of "The Hospital" should be addressed to the Editor, The Hospital, 28 & 29, Southampton Street, Strand, London, W.C., and should have the word "Nursing" plainly written in left-hand top corner of the envelope.]

### News from the Nursing World.

### THE RED CROSS.

Her Majesty Queen Victoria has been pleased to bestow the order of the Red Cross upon the Queen and the Crown Princess of Greece.

### OUR DISTRIBUTION FUND.

Our delightful task of distributing the contributions of useful garments sent us by our readers amongst the hospitals is over. Parcels have been sent to Charing Cross, the Metropolitan and the Great Northern Central Hospitals, the Hospital for Women and Children, Waterloo Road, the East End Mother's Home, the North London Consumption Hospital, and the Hospital for Epilepsy and Paralysis. The garments include men's shirts, vests, stockings, and comforters; and women's chemises, vests, bodices, petticoats, bed-socks, crossovers, and night-dresses; for the children, vests, petticoats, stockings, and a long dress, all of warm and useful materials. We thank all the contributors who have so kindly responded to our appeal. We must especially thank Nurse Ellen Clarke, whose box of delightful flannel garments, both for men and women, was, indeed, a splendid gift. The following is a list of those who kindly sent the acceptable contributions: Mrs. Delap, Miss Kate Saczbiovic, Nurse Willson, Nurse May, Nurse Anthony, Nurse Muir, Miss Mabel Thring, Miss Lockyer, Nurse Aston. "Aidyl," "Anon," Miss Good, and Miss Pritchard. We had pleasure in adding our own mite to the collection.

### OLD NURSES AT CHRISTMAS.

It is very pleasant to comply with the request of the Honorary Secretary of the Junius S. Morgan Benevolent Fund, and inform Pension Fund nurses of the happiness they have been the means of spreading this Christmas. At the last meeting of the Benevolent Fund it was found possible to devote a small sum to supplying some of the older nurses with the means to secure some little comforts at Christmas time. There are 36 pensioners now on the Benevolent Fund list, and out of these 16 were known to be specially needy and friendless. But thanks to the self-denial and generosity of those who have given so liberally to the Benevolent Fund, these sixteen old nurses have found friends at Christmas time, and each has received 5s. to provide good fare suitable to this season of rejoicing. Not only this. In one case a comfortable chair, and in another blankets, a warm petticoat, and a chair, have found their way to those sadly in need of such necessaries. It is delightful to think the pleasure these unexpected gifts have given. If any nurse who has joined in the good work of the Benevolent Fund is feeling sad and cheerless this Christmas time, surely the knowledge will be a consolation that she has brightened the lot of so many less fortunate than herself.

### INSTEAD OF A CHRISTMAS CARD.

In connection with the Prince of Wales's Fund, which we are quite sure has the hearty sympathy of our readers so closely connected with the hospitals, a clever scheme has been arranged this Christmas. Little albums bound in scarlet have been prepared to receive the Prince of Wales's stamps, and thus act as a collecting book for many annual subscriptions to the hospitals commenced in the Jubilee year for the sake of the Queen. The book contains a place for the signature, and also for a midget photograph of the subscriber. Several photographers have offered to photograph the owners of books containing a shilling and a half-crown stamp, free of charge. Now this scheme offers a variety of opportunities. First, the present of the little book can be made, instead of a Christmas card, at a cost of sixpence. If the recipient is a child, some other kind friend is sure to help in forming a little subscriber to the hospitals, and then there is further the interest and amusement of going to the photographer and having a photograph taken and fixed in the little book "all for nothing." The books can be had from chemists and booksellers, or direct from Messrs. Simpkin, Marshall and Co., London, and in them will be found the photographer's name. Little Prince Edward of York was one of the first children to subscribe to the Prince of Wales's Fund; and now we learn that many people in high circles are going to encourage charity amongst their children, and provide them with an interest in the Christmas holidays, by securing albums and a photograph of the young owners.

### "TRUTH'S" DOLLS.

The exhibition of dolls and toys in the Albert Hall during December 21st and 22nd was a sight to gladden the eyes and cheer the hearts of children and those who love them. It is quite out of the question to describe a tithe of the ingenuity and work bestowed upon these gifts intended to make the Christmas happy of sick children. One lady sent 400 dolls, another 300, and many sent batches of one or two dozen. Some of the large dolls were dressed in character; one represented the picture of "Truth," as seen on the cover of that journal. Unfortunately the classic lines of the drapery were marred by the fact that dolls, as a rule, are not anatomically graceful. The most charming, in our opinion, was a model of the Queen at the age of four, taken from a popular picture. But to say one was best among such a number of equal "beauties" is almost an impossibility. Whoever judges the respective merits in order to award the prize has no easy task. The most original and picturesque doll's house was made out of wine cases, and thatched with the straw bottle-sheathes; the windows were made from negative photographic glasses. No one who did not see it can imagine the ingenuity exercised upon it. The last scene in the "Little Minister," sent by the company performing the play at the Haymarket, attracted considerable attention. There were also several set scenes, the one least attractive represented the Queen and Crown-Princess of Greece visiting a camp hospital during the Græco-Turkish war. The ambulance bringing in a wounded and ghastly patient is hardly a suitable subject with which to amuse children, and their elders surely do not need too vivid a picture of the gruesome details of war. The prizes were handsome and valuable, whilst the 11,000 new sixpences for pauper children were exhibited in a glass case. They took up very little room considering their number and the happiness they were to give.

### MEDALS AND SERVICE.

Maidstone affords the second example of medals being awarded to nurses who have devoted themselves to the sick in times of epidemics. During 1894-5, Newport (Isle of Wight) was ravaged with typhoid, and when the plague was stayed the nurses were each given a medal, bearing on one side the arms of the town, with the legend, "In recognition of services rendered," and on the other "Typhoid Epidemic, 1894-95," and the name of the recipient.

### THE LITTLE EXTRAS.

The pence have an uncomfortable way of multiplying themselves into pounds. A little calculation and a few examples such as that afforded by the Bethnal Green Guardians would tend to make the position of those who hold the public purse more clearly understood by the nurses. The committee appointed for the purpose of inquiring into the dietary of the nurses recommended an extra allowance of half a pint of milk, two ounces of sugar daily, and sixpennyworth of fruit weekly. These items would cost about twopence a day for each nurse, and in her eyes it would probably be a trifle scarcely noticeable in the bulk of expenditure. But the proposed increase will, if carried, cost the ratepayers £350 a year. The matter is now under the consideration of the Finance Committee. We hope and expect that the nurses will have these extras granted them, as milk and fruit are certainly necessary articles of diet. But it must promote the mutual welfare and harmonious working of nurses, guardians, and ratepayers, if each body will take the trouble to understand and appreciate the difficulties of the others. For instance, when a nurse saves twopence by a little care and thought she makes up to the ratepayers that sum to spend on her comfort, and there is no telling how much money may be saved yearly from waste by a just appreciation of small economies.

### TRAINED NURSES OF ST. PATRICK.

The district nurse in Ireland carries healing in her touch for other woes than bodily sickness. At a sermon in St. Patrick's Cathedral in aid of the St. Patrick's Nurses' Home, Canon Tottenham, in speaking of the gratitude expressed by the poor towards this association, remarked that one old lady had said—"I would like to thank the Queen, but I believe that I will never get to speak to her in this world; but I look forward to be able to thank her in heaven." We are sorry to hear that this society, whose work is rapidly increasing, and whose nurses are so acceptable to the poor, is not strong financially. It is affiliated with the Queen Victoria's Jubilee Institute for Nurses, and this ought to insure liberal support.

### A SLIGHT MISTAKE AND A BIG DIFFERENCE.

Owing to a clerical error in the account given of the Christmas festivities at Brompton Consumption Hospital, it was stated that the "tree" was the gift of "Nurse," instead of the "Misses," Heddy. We are glad to learn that the Christmas entertainment is not left entirely to the nurses, as is only too frequently the case, and hope that next year other young ladies of leisure will follow the Misses Heddy's example, and lend their aid in the good cause of giving a happy Christmas to our hospitals.

### CHELTENHAM'S CHARITIES.

The Cheltenham District Nursing Association and the Needlework Guild both appear flourishing. More than enough members are now joining the guild to make up for the old ones who have been lost by various causes. The requisite sum to maintain a nurse has been raised by a sale of work, whilst grants of clothing have been made to charities. The good work of the Victoria Home is especially shown by the absence of puerperal fever in the maternity cases. A small lying-in ward has also been attached to the home, and pupils are trained for the L.O.S. diploma. There is a balance in hand, and the endowment fund stands at £2,000.

### COMMON SENSE AT SLOUGH.

The investment of the Diamond Jubilee Permanent Memorial Fund at Slough is at present under discussion, and Miss Brooke-Hunt, a lady who evidently takes much interest in the matter, and is ready to give generous support to a suitable scheme, writes a very sensible letter upon the subject. In the first place, she states that the actual wants of Slough at the present moment are an ambulance carriage to convey the sick to Windsor, a distance of three miles, a spinal carriage for children with hip and spinal disease, and an assistant nurse. She advocates the use of the money for these purposes, hoping that in due time a suitable home for the nursing staff may be built, for which she offers the site, and in which a couple of rooms could be set apart for special cases.

### COCKERMOUTH DISTRICT NURSES.

At the conclusion of the second year's work the committee of the District Nurse Fund, Cockermouth, are looking forward to reaping the advantage of affiliation with the Queen Victoria's Jubilee Institute. Affiliation with this institute ensures the association an honourable position in the nursing world, and it is by this means directly connected with and under the inspection of a body founded by the munificence of our Sovereign. Nurse Robinson, from the affiliated home in Harpurhey, near Manchester, began work in Cockermouth a few weeks ago. She has had four years' experience in a rough and poor district, and is very highly recommended by her late superintendent as a kind and conscientious nurse.

### THE YEAR'S RECORD.

The year's record of the Scottish branch of Queen Victoria's Jubilee Institute is one of hard work and of great success, marred by a deficiency in the income of £642. There are now 90 associations affiliated with the Scottish branch, employing 146 Queen's nurses, and 33 ladies completed their training during the year. The Council has received the first instalment of the Jubilee Commemoration Fund. It amounts to £300, and has been expended in training nurses, for whom there is a great demand.

### SHORT ITEMS.

The dance promoted by the Ladies' Committee in Aid of the Special Appeal Fund at Charing Cross Hospital, and held in the Empress Rooms, Royal Palace Hotel, Kensington, on the 9th inst., resulted in a sum of £240 being added to the Fund.

### Practical Aspects of a Nurse's Life.

By Sister Grace.

### XII.—NIGHT NURSES.

One must place high principle and conscientiousness first in the list of necessary qualifications for all nurses, but pre-eminently so for night nurses, because from the nature of their work they have many opportunities for neglecting their duties unseen, and unknown, if their own consciences allowed them. Night work as a rule is unpopular, and one must admit that a long course of it becomes monotonous, but that it is in any way unhealthy is, I think, purely imaginary. Where I was trained our matron put us on night duty if we were a little run down, because the work is so much lighter than by day; and certainly no nurse can consider herself properly trained who has not gone through a certain amount of night work.

In past years it was considered that anyone was good enough for this work in a hospital, and it was an event not uncommon to seek the aid of outside Gamps to supplement the inadequate supply of nurses inside the hospital available for this purpose; but now things are far different, and everyone recognises the importance of securing a nurse who is first-class in all respects.

She is practically alone at night and must rely on her own judgment, and act for the best on her own responsibility; true, there are generally one or two night sisters or a night superintendent in large institutions to apply to, but they cannot settle the many little difficulties that arise. She must be ready at a moment's notice to admit emergencies and accidents, and arrange for them for the best with perhaps inadequate help. How often one's heart has sunk on coming on duty to think of the many hours of comparative solitude before one, surrounded by so much suffering. Things always appear so much worse at night in the silence and darkness, but when morning comes one is surprised and cheered to find that instead of being worse, the patients are perhaps considerably better.

The habit of close observation is all important. The condition of the sick differs greatly in the night from that of the day, and the doctors and sister must rely on your account of that condition for their knowledge of the patient's state of health. Vitality is very low in the hours after midnight, and some cases often sink suddenly at that time. You must be very alert to mark changes of temperature, appearance, and respiration. Invalids are often extremely nervous at night and imagine many symptoms that do not exist; experience alone can teach you in these cases to eliminate the false from the true. Some night nurses have the valuable faculty of wooing sleep to the pillows of the most perversely insomnolent by many little manœuvres—turning the pillows, changing the bedclothes, putting a cool bandage on the head, giving a little hot milk, &c. . . . I found a most efficacious remedy among the old people was a small dose (hot) of peppermint water with sugar, which they assured me was most comforting "to the inside," a statement I was quite ready to take on trust.

### The Art of Giving an Intelligent Report.

Accustom yourself to give a succinct account of all your patients in your report, but avoid prolixity. It is an art by no means easily acquired. Confine yourself to facts, and do not indulge in impressions and suppositions unless you are distinctly asked to give your opinion; it is for nurses to give facts, and leave the doctor to draw his own conclusions. Keep a written note of all that occurs in the night for the sister, and should you unfortunately have forgotten anything, let nothing tempt you to suppress the fact.

Like most other difficulties of the same sort, a strong determination of will, will help you to strengthen your memory. Carefully review events several times in the course of the night to make sure that nothing is forgotten. If a nurse's memory is really defective, she is certainly unfit for hospital work, where it will be constantly and severely taxed.

Try to avoid a habit of calling the house-surgeon too frequently; it is a great temptation to do so when one is anxious and perplexed, but it is unfair to him, and the patients soon learn your weakness and hasten to take advantage of it, demanding his presence for the merest trifles.

Great circumspection in personal conduct is another point that I would impress upon you. A night nurse is practically alone, and in a peculiar position; you cannot, therefore, be too careful that nothing in your behaviour should cause invidious remark. Avoid everything which, though perhaps innocent in itself, is open to misconstruction. The class from which the majority of patients come have been accustomed from their youth to the seamy side of life, and so, poor things, it is not surprising that they are inclined to attribute low motives where none such exist. The danger to a nurse from the gossiping propensities of her patients is most common, I think, among the women, but your commonsense will tell you that there are others to be avoided in the men's wards. Never talk familiarly with them, or allow them to abate in the slightest degree the respect which is your due. Lastly, be careful in your intercourse with the house surgeons when making their rounds; it is a natural temptation to have a little chat with someone, amid so much solitude, but resist it. It is undignified, and may lead to much that is undesirable. Believe me, doctors greatly respect and admire a nurse who knows how to maintain a respectful and dignified reserve with them.

A good night nurse is a treasure to the sister; we ought, and I hope we do generally value them as we ought. How soothing it is to feel in going off duty, tired out with a long day's work, that we leave our patients in the hands of one who will work in the silent hours of the night, unseen and unnoted, heart and soul in the interests of the suffering. She receives but little recognition of her self-denying work; the only person who can give due gratitude for faithful service is the sister, and I hope they do not often forget to give honour where honour is due. In the course of eight years I had many night nurses, and by far the larger number I remember with pleasure and gratitude. They are scattered far and wide now, but if this should meet the eyes of any, I should like to tell them how affectionately I remember their zeal and patience with the sick, and the true and loyal devotion to myself, which helped me through many a dark hour.

### Probationers for Madras.

The following circular has been issued by the Surgeon-General of Madras: In view to the efficient training of women as nurses and ward attendants and of men as ward attendants for employment in private and up-country hospitals, the Government is prepared to admit probationers of these classes recommended by local or private bodies, such as the Lady Dufferin Fund Committee, into the General Hospital Training School for Nurses, on the understanding that such bodies shall arrange to defray the (1) pay, (2) ration-money, (3) clothing, travelling expenses, &c., of such probationers. Volunteers who are willing to pay all necessary expenses and who are approved by the Matron Superintendent will also be admitted. Quarters (for nurse probationers only) will, as far as possible, be provided by the Government at the General Hospital, but there is no objection to other arrangements being made by pupils subject to the approval of the Matron Superintendent. All applications should be made to the Matron Superintendent, General Hospital, Madras.

### Post-Graduate Clinics for Nurses.

By a Trained Nurse.

XXXVIII.—THE NURSING OF TYPHOID FEVER.

It is not necessary, of course, to give so wide a range of variety as that set forth in the last paper. I simply gave this dietary to show how many changes may be made, to the infinite benefit of the patient. I would like to remind nurses that malted foods and malt extracts are a most valuable form of food in fevers, but are generally not allowed in typhoids where much diarrhœa is present, since all malted foods are laxative. Dr. King Chambers says in fevers accompanied by dry mouth and throat he never allows farinacea such as arrowroot, &c., since the deficiency of saliva in these cases indicates that starch will not be digested. This, of course, does not apply to malted farinaceous foods, for in these the starch is already digested.

Three to ten ounces of alcohol are frequently given to typhoids during each twenty-four hours. Alcohol needs no digestion, and is therefore a very ready form of stimulation. If the doctor permit it is well to alternate brandy and whiskey, and the nurse should recollect that whiskey goes better with milk than does brandy and the latter better with cream.

It is a mistake to invariably give stimulants mixed with food, since the patient gets to loathe his spirit-flavoured nourishment. It is better to give the stimulant just before or just after the nourishment. Given a few minutes before food, alcohol often acts well in whipping up the digestive forces—like the soup before the dinner courses. The alcohol sets the mechanism of digestion going, and the patient is thus able to get at the nourishment contained in the food.

Brandy may be given, whipped up with slightly sweetened cream, the thorough whipping of the cream much adding to its digestibility and lightness. It should be placed for a few minutes on ice. Warm cream is apt to prove sickly.

In country places where ice is scarce, it is practical to know that by placing liquids in a porous earthenware vessel wrapped round with wet flannel, standing the jug by an open, sunless window, and keeping the flannel constantly wet, the contents of the jug can be kept at an ice-cold temperature. When supplies of ice are limited, the best method of preserving it is to put it in a flannel bag, enclosed in a larger flannel bag, and packed in feathers to the depth of three inches all round. Ice thus protected will last a very long time.

The dietary so far set forth for typhoid fever patients will furnish variety sufficient for the larger percentage of such cases. But there are always a certain proportion of typhoid patients who suffer from serious digestive disturbance, and for whom some of the formulæ given would not be suited.

Where digestion is much impaired, the ice-cream previously recommended may be made of peptonised milk, to which cream may be added proportionately to the gastric strength of the patient. This, too, will form a grateful dish to all patients whose dietary is restricted to peptonised milk—of which a sick person speedily tires.

Peptonised beef-tea, solidified with isinglass and iced, forms a delicious change from the everlasting sameness of liquids—the nurse always remembering that isinglass is infinitely preferable to gelatine in cases of digestive weakness.

Peptonised cocoa and milk forms a very tempting dish when solidified with isinglass and a little whipped cream poured over the shape. These creams and shapes should be freshly made every day, and I can assure any nurse who takes the trouble to manufacture such dainty little adjuncts to sick-room diet that her popularity will increase and multiply, and her services be in great demand. Every physician feels so secure of success when he recommends a nurse who is skilful at cookery.

Artificially-prepared milks are frequently of infinite value in cases of typhoid, and an admirable type of these, and one proving readily digestible, consists of 1 pint of good cow's milk boiled. Dissolve ½ oz of grape or milk sugar, and ½ oz. of gelatine in half a-pint of hot barley water. Add this to the milk, the whole proving a light, digestible, and pleasant food.

Dieting in typhoid fever depends largely on whether constipation or diarrhœa be present. If the latter, beef teas and meaty infusions will not be allowed. But raw meat juice is frequently given where excessive diarrhœa is going on, as this seems to have no laxative effect whatever.

With regard to constipation, nurses are too apt to attribute this to a milk diet, when in reality it arises most from loss of nerve power in the bowels through inflammation, from depressed vitality, and partly, too, from the absence in the intestinal tract of the normal amount of solid residuum which exercises a certain stimulus on peristaltic action. The diet in typhoid is planned so that the greater part of the nourishment is digested in the stomach, leaving little residue to be dealt with in the intestines. The flannel kummerbund, so essential in typhoid, has some action on the relief of constipation by keeping up the warmth and vitality of the abdomen. If the doctor allows a small cup of black coffee, this without sugar taken early in the morning often relieves the constipation, which, if not relieved in other ways, will necessitate the use about every third day of an enema. This should consist of a pint of thin warm gruel or barley water. Glycerine injections are not usually ordered in typhoid as these are apt to cause irritation of the lower bowel.

It is far better, of course, if constipation can be overcome by dieting. Malt extract, aërated waters, and beef-tea all act well in this direction. Buttermilk is occasionally ordered because of its laxative properties, but as a rule patients do not much like it. It is nutritious and digestible, since the casein of the milk is coagulated and finely divided. But for typhoid, it needs boiling and straining, by which time it has lost much of its nutritive value.

Buttermilk, which is often employed in obstinate nausea and vomiting and chronic gastric catarrh, should be used fresh. After forty-eight hours' standing it is apt to become unwholesome through the development of lactic acid.

Where nausea and vomiting occur in typhoid, and if hæmorrhage be severe, it may become necessary to resort to nutritive enemata, for a time giving nothing whatever by the mouth. I would like here to say something of nutrient enemata, for I frequently find certificated nurses administering so-called "nutrient enemata" of raw milk and uncooked arrowroot, or of beef-tea thickened with cornflour. I am perfectly aware that for this their training school is more or less responsible. At the same time, it is rather hard on a sick person to rely upon such an enema in times of crisis. Apart from science, commonsense readily points out to us that in a normal state of affairs by the time food reaches the bowel it has undergone many digestive and chemical changes. Under such conditions the large bowel is able to absorb a certain amount of the nourishment from such changed food. But the raw material injected—without previous treatment—presents embarrassing and unusual conditions to the bowel, which is able, therefore, to extract very little nourishment from the crude material. Sir William

Roberts favours the addition of liquor pancreaticus to nearly every form of nutrient enemata, a dessert-spoonful of this being added to every injection.

Starchy substances used in nutritive enemata should invariably be predigested, as they are thus more easily absorbed, and consequently less rectal irritability is produced. Recent experiment seems to show that 58 to 70 per cent. of fluid egg albumen may be absorbed from the rectum without peptonisation if salt be added in the proportion of ½ drachm to each egg.

An excellent enema is formed of one tablespoon of Mellin's Food, one teaspoon of cream, and one teaspoon of Valentine's meat juice. Warm water to bring mixture up to 1½ ounces. Some opium may be added, but only by medical instructions. One teaspoon of meat peptone in a dessertspoon of peptonised gruel and one dessertspoon of double cream is a good formula. The bowel needs occasional washing out with tepid water during the continuance of nutritive enemata, and when the bowel becomes irritable and easily rejects the injected fluid, some lint firmly and tightly bound round the base of the tube of the syringe forms a kind of support, thus helping the bowel to retain the fluid. A firm lint pad may also be held close to the anus for ten minutes after the withdrawal of the tube.

The abdominal distension or tympanites of the bowels which frequently occurs in typhoid is often met by the doctor ordering an enema of oil of turpentine, or turpentine may be given by the mouth. Sometimes a rectal tube is introduced and left *in situ* to relieve the flatus. But if the bowel be in an irritable condition this may have to be discontinued through its tendency to cause diarrhœa.

The nurse may apply hot fomentations or ice-cold compresses to the abdomen, either of which will relieve the pain and discomfort of distension. Occurring in the early stages of typhoid fever, distension is an unfavourable symptom. Cradling should be employed to keep off the oppression of the bed-clothes, and some care should be exercised that undue quantities of fluid be not taken. Some of the semi-solid forms of nourishment already recommended may prove favourable in these cases of painful tympanites.

### Royal British Nurses' Association.

The special meeting of the members of the Royal British Nurses' Association, held by command of Princess Christian, the president, at the rooms of the Royal Medical and Chirurgical Society, 20, Hanover Square, W., on Friday, the 11th inst., to consider the new draft bye-laws, was an object lesson in the gentle art of obstruction. From start to finish a very small minority showed how effectively an organised opposition could obstruct business.

There was a large attendance, between 260 and 270 persons being present. Of these the opposition, numbering about 20, were formed up in a compact little body in the front seats on the right hand side of the room.

The Princess Christian, after reminding the meeting that the new bye-laws had been discussed and passed by the Executive Committee and approved by the Council, emphatically stated that whatever she, or those who worked with her, did, they were actuated by but one wish, the good and the unity of the Association. (Applause.)

Sir Dyce Duckworth took the chair.

The Hon. Medical Secretary (Mr. Fardon) formally proposed the adoption of the new bye-laws and the submission of them to the Privy Council. This was formally seconded.

Amendments to the bye-laws were then called for, the ruling of the Chairman that those amendments of which notice had, in response to the request on the agenda paper, been sent in prior to the meeting, would be taken first, being objected to by Dr. Bedford Fenwick on the ground that this was not in the Charter.

In response to the Chairman's request, Dr. Wethered moved that the bye-laws be amended so as to enable the first annual meeting after the sanction of the bye-laws by the Privy Council being held at such time as the President might appoint instead of, as usual, in May or June. This amendment brought Dr. Hugh Woods to his legs, as he was of opinion that there was a great deal behind this amendment. Why, he asked, should the Association wish to alter the date of the annual meeting at anybody's pleasure but its own? The Hon. Medical Secretary explained that it was in order that the first annual meeting might be held as soon after the bye-laws were sanctioned as possible, so that the new Council and the new Executive Committee might get to work without delay. "In other words," said Dr. Hugh Woods, "I am to understand that by these bye-laws it is intended to turn off the Executive Committee certain individuals, and you are anxious to do it as promptly as possible." The amendment was put to the meeting, and carried.

Another amendment by Dr. Wethered, altering the date of the meeting of the Executive Committee from July to a day not less than fourteen and not more than twenty-eight days after the annual meeting, elicited the plaintive cry from Dr. Hugh Woods that really, on the spur of the moment, he could not take in what these amendments meant. These amendments should not have been sprung upon this meeting, but should have been considered in committee. After a slight excursion into other matters on the part of the opposition, the amendment was put and carried.

A third amendment by Dr. Wethered asking that the words, "as amended," be inserted in Mr. Fardon's resolution, so that it read that the "bye-laws as amended be approved," called forth further protests from Dr. Bedford Fenwick.

After it was carried, Dr. Hugh Woods asked that the amendment should be read, as he really did not understand it, whereat a member pertinently remarked, "Then why did you vote against it?"

Dr. Biernacki moved an amendment, putting in the hands of the Association itself rather than in the hands of the Executive Committee, the power of defining the qualifications to be possessed by nurse candidates for membership.

The Hon. Medical Secretary said that, while thoroughly appreciating the moderate way in which Dr. Biernacki had put his proposition before the meeting, he could not accept the amendment, as he failed to see that Dr. Biernacki had produced anything which was really in principle contrary to what the committee had placed before the members in those bye-laws.

Dr. Hugh Woods said he did not want to waste the time of the meeting, a remark which was greeted with laughter (indeed, from here to the end of the meeting each successive appearance of Dr. Woods seemed to tickle the meeting immensely), but there was this difference in the two proposals, that it was a question of whether the members were to have the power of saying what a nurse is, or whether the Executive Committee was to have that power.

Mrs. Bedford Fenwick, after disclaiming all responsibility for the bye-laws, although she was a member of the Executive Committee, said that Dr. Hugh Woods had pointed out the vital difference between the two proposals, and then branched off into a dissertation on the admission of asylum attendants to the register. In spite of her protests that she was quite in order in doing so, because she *knew* she was in order, the CHAIRMAN gently, but firmly, brought her back to the subject under discussion.

The amendment was put to the meeting, and the CHAIRMAN declared it to be lost, upon which Dr. BEDFORD FENWICK asked, amidst interruption, that the numbers be taken.

Dr. BIERNACKI at once rose and said that it was he who had moved that amendment, and that he was quite satisfied with the ruling of the Chairman.

Dr. BEZLY THORNE was next called upon, and his rising was greeted with hisses from the opposition. This gave him an opportunity of scoring. Taking advantage of a remark previously made by Dr. Bedford Fenwick, when one of his attempts at obstruction had been greeted with hisses, that hissing and any attempt to disturb the meeting were undignified, Dr. Bezly Thorne turned the tables by quietly telling his opponents that a very high authority had pronounced against this form of amusement. Dr. Bezly Thorne's amendment provided for the retirement of all the matrons, *ex-officio* or otherwise, triennially. The principle, he said, was one which had been set up in the Association by Dr. Bedford Fenwick himself as far back as November, 1891.

Miss GRACE GORDON seconded.

Dr. BEDFORD FENWICK complained that Dr. Bezly Thorne had not given the meeting both sides of the matter connected with the retirement of the matrons. He held that the Association had, in depriving the leading matrons of permanent seats, acted dishonourably towards persons who had aided the Association in its infancy so soon as the necessity for that aid had disappeared. He took upon his own shoulders the discredit of the bye-laws passed when the Charter was granted not being so definite on the point of granting these seats to the matrons as those in force before that time. He could only plead the short time he had for drafting those bye-laws in time for their submission to the Privy Council.

The HON. MEDICAL SECRETARY regretted that he could not accept Dr. Thorne's amendment, as its practical effect would be to limit the number of *ex-officio* representatives. It was because he thought that the matrons of the great training schools, who would form the *ex-officio* matrons on the Council, represented all that was best and most progressive in what appertained to nurses, and the training and education of nurses, and so would form a nucleus of advice which the Association could fall back on, that he could not accept the amendment. It would be inconsistent with all he had said if he did not do all he could to get those matrons permanently upon the Council.

The amendment was lost.

When the amendments which had been sent in prior to the meeting had been exhausted, the CHAIRMAN asked whether any member had other amendments to propose.

Dr. BEDFORD FENWICK said he had with respect to the first bye-law appointing a patron.

The CHAIRMAN asked him to hand in his amendment in writing.

Dr. FENWICK ignored the request, and proceeded to make a speech on the point, in which he stated that the bye-law was contrary to the Charter, and was therefore *ultra vires*.

The CHAIRMAN again requested Dr. Fenwick to hand in his amendment in writing.

Dr. Fenwick again ignored the request, and

Dr. HUGH WOODS proceeded to second something. He said it was stated that the draft bye-laws had been laid before eminent counsel, and he thought some explanation was due from the officials of the reason for their allowing an illegality in the first bye-law.

The HON. MEDICAL SECRETARY said he would be very happy to give that explanation. There was nothing in the Charter forbidding the appointment of a patron, and the Association was within its rights in doing so.

The words of the amendment had by this time reached the CHAIRMAN, who said that it was to the effect that the bye-law be deleted.

This was put to the meeting and lost, and the bye-law was accepted.

Dr. BEDFORD FENWICK and Dr. HUGH WOODS, followed this up by proposing that Bye-laws II., III., IV., VII., and IX. be deleted.

These were held not to be amendments, and a large amount of time was wasted in arguments as to the proper way of carrying on the business, these two members holding that each bye-law should be proposed and seconded separately in order to carry out the provisions of the Charter, and the CHAIRMAN holding that the point was sufficiently covered by the resolution proposed at the beginning of the meeting by Mr. Fardon, it only being necessary that amendments should be proposed as the bye-laws were gone through seriatim.

Sir JAMES CRICHTON BROWNE could not see that Dr. Bedford Fenwick and Dr. Hugh Woods had the monopoly of knowing how to conduct the business of the meeting, and he asked the meeting to support the chair. (Applause.)

Dr. HUGH WOODS said that if the bye-laws were not individually proposed and seconded, the matter would be brought before the Privy Council.

The motions that the five bye-laws be deleted were in turn put to the meeting and in turn lost.

On an appeal from Mr. FARDON that business should not be obstructed,

Dr. HUGH WOODS said that to hasten business he would come to the most important point in the whole of the bye-laws, the exclusion from the Executive Committee of the president of the Incorporated Medical Practitioners' Association. He moved that the Executive Committee continue as before. The Medical Practitioners' Association had helped the Nurses' Association at the first, and, as a sort of compensation for that help, the Nurses' Association had agreed to make the president an *ex-officio* member of the committee. Now that they found the president was an independent member, who would not do as he was told, they wished to get rid of him. This he held to be a deliberate breach of faith and a deliberate insult to the medical profession.

Miss BREAY seconded Dr. Hugh Woods. In the course of her speech, which she read, she said that the only justification the Association could now offer for the breach of faith alluded to by Dr. Woods would be that the promise was not made over a sixpenny stamp. The meeting, she said, was asked to remove from the bye-laws and from the Executive Committee the name of Mrs. Bedford Fenwick, the founder of the Association, a remark greeted with cries of "Why not?" and "She is not the founder."

The CHAIRMAN here interrupted the delivery of the address, and thought Miss Breay should not go into personalities.

Miss BREAY, however, continued to read her address, and finally asked the meeting to note how, in that corporation of nurses, the nurses were that day represented on the platform. Only one matron had a seat there, said Miss Breay, "and that lady's influence had always been at the disposal of the officials."

This uncalled-for attack was received with a storm of hisses, on the subsidence of which

The CHAIRMAN said he could not allow Miss Breay to proceed in that manner. He stigmatised her language as highly improper and most indecent, and especially so as it had evidently been penned before the meeting was held. (Loud applause.)

After some further remarks from Miss BREAY, the meeting declined to hear her any longer, and she resumed her seat, hoping that this would be an example to the press of what happened when persons expressed their opinions in the Association.

By this time the meeting had become thoroughly tired of the continuous obstruction, and Sir CHARLES GAGE BROWN moved that, as everybody had already had an opportunity of considering the bye-laws, they be put to the meeting *en bloc*.

This was hotly opposed by Dr. HUGH WOODS and Dr. BEDFORD FENWICK, the former of whom strode up to the platform and declared everything was intended as a deliberate insult to himself, or to Dr. Bedford Fenwick, or to Mrs. Bedford Fenwick, or to someone else. He also lamented the fact that so far his efforts as a peacemaker had been met by an attempt to remove him from the governing body—(laughter)—and threatened that if the meeting passed the bye-laws there was plenty more fighting ready prepared, and the Association would never get out of hot water.

The meeting, weary of the ceaseless obstruction, declined to hear any more, and Sir Charles Gage Brown's resolution was put and carried by a large majority, about 20 hands being held up against it. A poll was demanded, but was not taken.

A resolution was carried providing that from the date of their approval by the Privy Council the new bye-laws come into operation, and

The meeting shortly afterwards broke up.

### In a Christmas Library.

### MISS BOBBIE.

This story reminds one somewhat of Miss Mouse and her boys. But Miss Bobbie is a nicer little person—a red-haired Irish-Australian lass, and her boys are much more delightful squires of dames than were those attendant on Miss Mouse. The Australian setting of the story adds much to its interest, and throughout it is told in a charming womanly fashion, which will go straight to the hearts of young—or any other—readers.

### BEETON'S CHRISTMAS ANNUAL.

This is a capital collection of stories, "A Professional Beauty," by E. P. Train, being smart writing—and smart light reading, too. An admirable story by the author of "Miss Bobbie," tells of the efforts of some small philanthropists to transform a street waif into a "real lady." Beeton's is a valuable shillingsworth.

### WINDSOR MAGAZINE CHRISTMAS NUMBER.

The "Windsor" Christmas Number is a wonderful production. In addition to stories by Ian Maclaren, Guy Boothby, Zangwill, and L. T. Meade, a novel by Grant Allen, entitled "The Scallywag,", is given away. This is enterprise and generosity indeed, for the Christmas number in itself would furnish a whole week's reading. The illustrations are excellent, and the standard of the letterpress extremely good.

The publishers of "The Singer of Marly" are Messrs. Methuen and Co., and those of "The Highway of Sorrow" are Messrs. Cassell and Co. Those of "The Outlaws of the Marches" are Messrs. Fisher, Unwin, and Co. By an error the names were wrongly quoted last week.

### Our American Letter.

THERE is not much of special interest in the American nursing world just at the moment, but an account of the mothers' classes at the Nurses' Settlement in New York is full of information and practical suggestions. There are already nurses' settlements in London, and it seems probable that the example of New York in the matter of mothers' classes could be followed with a considerable degree of profit.

The general idea is to assemble mothers of the poorer classes together and teach them elementary domestic hygiene and cooking in a friendly way, just as mothers' meetings are convened to teach them moral truths and sewing. The manner in which this is done is happy. Upon the appointed day the nurse in charge receives the poor mothers with all the courtesy due from a hostess to her guests. Then follows some simple demonstration, such as how to make a bed or a poultice, how to cook simple dishes, or how to wash and dress the baby. This over, the company adjourns to the tea-room, where the table is nicely laid with pretty china and flowers. The rough and unkempt guests speedily acquire a certain amount of courtesy in their demeanour one towards another, and enjoy themselves amazingly. The evening is spent in simple games. Sometimes, a friend will play or sing for them; but, whatever it is, the mothers so thoroughly amuse themselves that a gentle reminder from their hostess to the effect that hungry husbands require supper is necessary before they take their departure.

The mothers who look forward with delight to this solitary weekly social dissipation are divided into two classes. The one consists of those that must have their instruction delivered in Yiddish—the dialect of the modern Jews, the other of women who can speak English. The latter class is again roughly and amusingly divided into those who wear hats and those who do not. Foreigners pay more attention to a coiffure and a jaunty apron, whilst elaborate headgear is the favourite vanity of their more anglicised sister.

The demonstrations are arranged in courses and are only held during the winter months. Occasionally, one or another of the students will ask a question and a demonstration is then inserted in the programme to explain the answer. During the summer-time the mothers bring their mending and their babies, who are allowed to play in the sand, whilst some one reads a story or the women pass the time talking over the endless questions associated with the problem of maternity and poverty.

### Where to Go.

DECEMBER 30TH. — Brompton Consumption Hospital: Christmas entertainment, half-past six p.m. Moorfields Eye Hospital: Christmas tree, half-past four to seven p.m. St. Mary's Day Nursery and Hospital for Sick Children, Plaistow, E.: Children's entertainment, three to six p.m.

DECEMBER 29TH.—London Hospital: Children's Christmas tree and Punch and Judy show, three p.m. East London Hospital for Children, Shadwell, E.: Christmas entertainment at three p.m.

THE NEW GALLERY.—An interesting exhibition of ancient and modern works by artists of the British and Continental schools is now open at the New Gallery from ten to six daily.

DECEMBER 31ST.—Moorfields Eye Hospital: Christmas entertainment at half-past seven p.m.

### "THE HOSPITAL" CONVALESCENT FUND.

WE are glad to see that our readers do not forget THE HOSPITAL Convalescent Fund, and we have much pleasure in thanking "Perthshire" for her kindly gift to it of 5s.

### Some Improvements at the London Hospital in 1897.

As we foretold, the year 1897 has been one of progress at the London Hospital. A great deal has been done towards replacing the antiquated furniture in the wards by that which is more up to date and better adapted to modern requirements. New bedsteads have been supplied throughout the building, of an excellent pattern for hospital use. These bedsteads are 27 inches high and 3 feet wide, and they can be adapted to a fracture case by the addition of a simple appliance, which is a great convenience. This arrangement enables the beds to be kept of uniform height and appearance even throughout the accident wards. Apparently, this height of bedstead enables both short and tall women to nurse their patients with greater facility than with the old-fashioned ones; and, for the process of ward bed-making, these beds are a great saving of fatigue to the nurses. The heads of the bedsteads are removable to facilitate the giving of anæsthetics. Linen pillow-cases have been supplied throughout the hospital, so that the pillows can be moved and changed to suit the needs of the case and the comfort of the patient, instead of being folded away in a solid lump under the bottom sheet, as was formerly done. The hospital laundry has been enlarged, although we understand that the available space for laundry work remains very inadequate to the needs of this large institution.

One improvement has a satisfactory way of making others to follow in its train. The new bedsteads emphasised the long-felt want of fresh lockers throughout the building. These are too costly to be provided except by gradually supplying a few at a time for the different wards. The cost even of small improvements is a great consideration when every article of furniture desired for the patient has to be multiplied by 800! An excellent form of bed-table has also been introduced. It runs smoothly on castors, and stretches across the bedstead without resting on the bed or patient, as is the case with all the old-fashioned patterns, detracting much from the patient's comfort in using them, by keeping the patients in a constrained position, and running the risk of being upset by any sudden movement. The new bed-tables are very popular. A firm, satisfactory pattern of bed-rest also has been supplied, wide enough to keep the pillows in place, and it adds immensely to the comfort and convenience of patients.

In the surgical wards glass and metal tables, glass dressing stands, receivers, and various appliances used for surgical dressings are being supplied by degrees, and the improved lotion stands are a practical convenience and a saving of labour to the nurses in the doing of dressings or when minor operations are done in the wards. The old-fashioned metal lampstands and inkstands have been superseded by others of the same convenient shape, but made in porcelain, and so far the fears entertained that these might prove too brittle for daily wear and tear have not been justified by experience. The time-honoured custom of nurses and probationers spending half the night in polishing lamps and inkstands is at last abolished, to their great satisfaction and to the advantage, we feel sure, of the patients.

Several of the ward floors have been covered with a suitable pattern of carefully laid down indurated linoleum. This is found a very satisfactory material for covering ward floors. It deadens the sound of the traffic, is very easy to sweep and clean, and has a good appearance. Other wards will be included in this arrangement when the necessary funds are forthcoming.

The ever-increasing proportion of acute cases at this hospital has rendered it absolutely necessary to provide the surgeons with additional accommodation for emergency operations. Pending the building of a new operating theatre of the same size as the existing theatre, and the necessary accommodation appertaining to it, one of the wards at the top of the building has been transformed into a new operating room, which proves very well adapted to its purpose as a temporary measure. All these and other minor improvements have been provided for the direct benefit of the patients.

The hours on duty of the whole nursing staff have been reduced. We record with pleasure that during the greater part of this year sisters at the London Hospital, besides the daily two hours off duty, have had half a day every week, in addition to a whole Sunday off every fortnight; thus, every alternate week each sister may absent herself from the hospital from two p.m. on Saturday until eleven p.m. on Sunday. All staff nurses and probationers on night duty have had a half-day off once a fortnight and a whole Sunday once a month, one of the half-days and the Sunday being arranged so that a nurse may be away from the hospital from two p.m. on Saturday until ten p.m. on Sunday. We are glad to learn that after next month the off-duty time of staff nurses will be increased to half a day once a week. Probationers on day duty have, up to the present, had a whole day off once a month; but after next month we understand that they will have the equivalent to half a day off every week in the shape of a whole day off duty every fortnight. And a whole day off at the London now means a whole day, and not, as heretofore, a whole day less two or three hours' work in the wards in the morning. In addition to this it has been arranged that probationers get four separate hours out of the wards every week for attending lectures and study classes, so that this time has no longer to be deducted (as was formerly the case) from the daily two hours off duty. We congratulate the nursing staff of the London Hospital on a time-table which should make their lives brighter and happier, and which should go a long way towards making them refreshed and invigorated for their arduous work, and we hope that it may soon come to represent the minimum in every well-regulated hospial.

### Appointments.

### MATRONS.

THE NEW HOSPITAL, ZOMBA, BRITISH CENTRAL AFRICAN PROTECTORATE.—Miss Lucy Agnes Harrison and Miss L. B. Stowell sail next week for Zomba to take charge of the new hospital there now in course of construction. This hospital is designed for the use of the officials stationed in the forts scattered throughout the district. Miss Harrison was trained at St. Bartholomew's Hospital, where she was gold medalist of her year, and has lately been assistant matron of the Royal South Hants Infirmary, Southampton. Miss Stowell was trained at Guy's Hospital, where she has also been sister of the Patience Ward. The appointments have been made by the Foreign Office, and the salaries are £135 per annum each, with house and outward and return (after two years) passage.

CUMBERLAND INFIRMARY, CARLISLE. — Miss Elizabeth Gordon Wemyss, who resigned her office of matron at the Bedford General Infirmary and Fever Hospital last September, after five years' work, was appointed Matron of the Cumberland Infirmary on December 16th. She was trained at the Middlesex Hospital, and was matron at the Jaffray Hospital, Birmingham, for nearly two years.

HOSPITAL SAMARITANO SAO PAULO, BRAZIL.—Miss Lillian Lees has been appointed Matron of the above hospital. She was trained at the Manchester Children's and at King's College Hospitals, and has since been ward sister at the Lewisham Infirmary and nursing sister in the late war in Greece.

LIVERPOOL CHILDREN'S INFIRMARY.—Miss P. K. Plunket, who was trained at King's College Hospital, was appointed Lady Superintendent of this infirmary on December 16th, 1897.

### Common Sense.

Many discussions are taking place just now as to the best means of providing for age amongst the attendants on the insane. It appears that the actuary (Mr. J. A. Robertson, C.A., F.F.A.), who has been consulted upon this subject by the Scottish Committee of the Medico-Psycological Association, recommends some arrangement being made with the Royal National Pension Fund, giving as his reasons for this advice that (1) the Fund is well established (2) and has a large donation bonus fund, no less a sum than £5,000 being divided in 1892 amongst the first 1,000 nurses that joined. As all nurses are not aware of their full privileges with regard to this Fund, and for the benefit of asylum attendants who may not have had the opportunity of seeing a memorandum from Miss Evans in the *Asylum News* for November 15th, we reprint it.

"To start a new benefit society would mean a considerable sum as a reserve deposited in a bank, or it would have to be arranged that members could not make any claim on the society until the reserve reached a certain amount.

"Offices would have to be rented in London or elsewhere, and paid offiicals found, such as secretary, clerks, and auditors.

"All this would take years to inaugurate and consolidate on a sound mercantile basis, and it would involve a series of preliminary meetings at which attendants, nurses, and their delegates would find it difficult to be present.

"Why, therefore, not join the Royal National Pension Fund, which has a well-organised staff, and has two branches —one for pensions and one for sick fund? This fund has a large reserve and a very large number of members, so that there is no danger of collapse.

"If the Lunacy Act Amendment Bill appears again next year, and if the Worcester Asylum suggestion about pensions be rejected, and asylum officials placed under the same conditions as workhouse officials, it would mean such a long service that very few of us could stand the wear and tear.

"Even if the Worcestershire proposal were adopted, or the old condition of things adhered to, the pension which a nurse or attendant receives is not large, as it can never exceed two-thirds of salary and allowances. Therefore, every asylum nurse and attendant should join the Pension Fund, and then the two pensions together would provide sufficient to live on.

"The above fund is not so well known among asylum staffs as it ought to be, and many have an idea it is only for hospital nurses, and that asylum officials are not eligible as members.

"I advise every member of the Assocation of Asylum Workers to write to the secretary for a prospectus, and see for themselves what benefits are offered to all who join the Royal National Pension Fund for Nurses (Male and Female).

"The address is 28, Finsbury Pavement, London, E.C."

### Novelties for Nurses.

### OATMEAL STOUT.

(Lewis and Barker, Wellow Brewery, Grimsby.)

Our attention has recently been drawn to this new departure in the brewing of malt liquors, and from trial and examination which we have made we are able to pronounce a judgment most favourable to Messrs. Lewis and Barker's oatmeal stout. This stout, though in many respects resembling the usual varieties, differs from them in a few essential points. In the manufacture, in addition to the usual malt and hops, there is superadded a certain quantity of Scotch oatmeal grist. The stout has, in consequence, a characteristic flavour, which is highly agreeable. The colour is much lighter than that of the usual varieties. In our experience this stout is better tolerated by the delicate stomach than the stronger and heavier forms; it has less tendency to produce flatulence, and is a nutritive food as well as a stimulant beverage. For invalid and nursing mothers it will be found less soporific and heavy than the usual forms.

### Social Amenities of the Royal British Nurses' Association.

### The Conversazione.

The annual conversazione of the Royal British Nurses' Association on Wednesday, December 15th, was largely attended and very successful. The handsome rooms of the Institute of Painters in Water Colours, Piccadilly, were filled with animated guests, the majority of whom wore the picturesque garb of their calling, and were without doubt *bonâ fide* nurses, thoroughly enjoying the social opportunities of the occasion. Various entertainments were provided. The electrophone occupied the table of a small room, and it was most amusing to see the faces round it, their owners listening to the music at the Savoy or Gaiety. In the larger hall an interesting musical programme was given, followed by Mr. Devant's excellent and well-known conjuring and shadowgraphs. The refreshments were highly appreciated.

### The Concert.

On the 20th inst. Miss Maud Danks gave a concert in St. James' Hall, Piccadilly, on behalf of the benevolent fund of the same society. The hall was well filled. The programmes, which were distributed by nurses in uniform, contained the disappointing announcement that Mrs. Clement Scott, on account of the dangerous illness of her husband, was unable to give her promised recitation. The instrumental music was brilliant and sympathetic. Messrs. Ross and Moore at the two pianos and Mr. Percy Such on the violincello were all applauded, and Miss Danks and Miss Steward both received a cordial welcome. The incident of the bouquets was pleasing. Miss Danks, upon being recalled at the end of her first song, had a couple of pink posies handed up to her, which were immediately followed by a number of white ones. The young lady was overwhelmed in two senses—she could not carry away all her treasures, and was evidently unexpectedly delighted with these tokens of recognition for her services. Mrs. Plowitz-Cavour sang some German ballads very sweetly, and Mr. Ffrangcon-Davies sung two songs and gave two musical recitations—called cantillations—in a powerful and finished manner. Doubtless the benevolent fund will be much enriched by the exertions of the artistes and friends who have so kindly given time and service for this object, and especial thanks should be given to Miss Foggo Thomson, who inaugurated the concert.

### Everybody's Opinion.

[Correspondence on all subjects is invited, but we cannot in any way be responsible for the opinions expressed by our correspondents. No communication can be entertained if the name and address of the correspondent is not given, as a guarantee of good faith but not necessarily for publication, or unless one side of the paper only is written on.]

### AN UNSOLICITED TESTIMONIAL.

We are glad to publish the following extract from a letter received from "An Old Probationer" in regard to the unfounded charges lately made against Nurse Warrener: "I was trained under her and had ample opportunity of seeing her care and kindness to all under her. For the patients' happiness and comfort she spared no trouble or pains. I am proud of my matron, and thankful for the thorough training she gave me."

### A DISTRICT NURSE'S STIPEND.

"E. C." writes: I should like to thank the editor of The Hospital for saying that he considers "a salary of less than £65, with lodgings, too low." I have £65, without lodgings, and although I make ends meet I certainly cannot make any provision for the future. It would be much better if committees and those who employ district nurses would provide them with comfortable lodgings. Surely it is not unreasonable to ask that a nurse who is willing to work at all hours and in all weathers for the benefit of her patients should be looked after and made comfortable when her work is done.

### For Reading to the Sick.

"REJOICE in the Lord alway."—*Philippians* iv. 4.

**Verses.**

O how glorious and resplendent,
Fragile body, shalt thou be,
When endued with so much beauty,
Full of health and strong and free,
Full of vigour, full of pleasure,
Thou shalt last eternally!

Now with gladness, now with courage,
Bear the burden on thee laid,
That, thereafter, these thy labours
May with endless gifts be paid;
And in everlasting glory
Thou with joy mayst stand arrayed.
—*Latin Hymn.*

Awake, sad heart, whom sorrow ever drowns;
Take up thine eyes which feed on earth;
Unfold thy forehead, gathered into frowns;
Thy Saviour comes, and with him mirth.

Arise, sad heart, if thou dost not withstand,
Christ's Resurrection thine may be;
Do not, by hanging down, break from the Hand,
Which, as it riseth, raiseth thee.
—*George Herbert.*

Renounce joy for my fellows' sake? that's joy
Beyond joy; but renounced for mine not theirs!
Why, the Physician called to help the sick,
Cries, "Let me, first of all, discard my health!"
No, Son! the richness hearted in such joy
Is in the knowing what are gifts we give,
Not in a vain endurance not to know!
Therefore, desire joy, and thank God for it.
—*Browning.*

Oh! for the joy Thy presence gives!
What peace shall reign when Thou are here!
Thy presence makes this den of thieves
A calm, delightful house of prayer. —*Cowper.*

But oh! the folly of distracted men,
Who griefs in earnest, joys in jest pursue,
Preferring, like brute beasts, a loathsome den
Before a Court, e'en that above, so clear,
Where are no sorrows, but delights more true,
Than miseries are here! —*Herbert.*

Life's inadequate to Joy. —*Browning.*

I looked for Evil, stern of face and pale;
Came Good, too fair to tell.
I leaned on God when other joys did fail;
He gave me these as well. —*S. Williams.*

**Reading.**

"Ye shall rejoice in all that ye put your hand to."—*Deut.* xii. 7.

Nothing glorifies God so much as joy. It is self which has marred this joy. It is humility above all other things which weakens or snaps asunder the holdfasts of selfishness. A lowly spirit is of necessity an unselfish one. Humility is a perpetual presence of God, and how can self be otherwise than forgotten there? A humble man is a joyous man. There is no worship where there is no joy. For worship is something more than either the fear of God or the love of Him. It is delight in Him.—*F. W. Faber.*

Let us serve God in the sunshine, while He makes the sunshine. We shall then serve him all the better in the dark, when He sends the darkness. It is sure to come. Only let our light be God's light and our darkness God's darkness, and we shall be safe at home when the great nightfall comes.—*F. W. Faber.*

### Minor Appointments.

TORBAY HOSPITAL, TORQUAY.—Two Charge Nurses have recently been appointed to this hospital: (1) Nurse Kirtley, late charge nurse at Winchmore Hill, who was trained at Mill Road Infirmary, Liverpool; (2) Nurse Harding, who was trained at the Royal Albert Edward Orphan Infirmary, Wigan, and who has lately been charge nurse at the County Infirmary, Brecon. Both ladies are certificated and hold excellent testimonials.

ST. MARY ABBOTT'S INFIRMARY, KENSINGTON.—Miss E. Nalder, who has lately been sister of the maternity wards of this infirmary, has been appointed Sister of the male wards. Miss Nalder was trained at Leicester Infirmary, and after working at the Clapham Maternity Hospital, took the L.O.S. certificate. She was afterwards for a time engaged in private work.

WEST RIDING ASYLUM, MENSTON, YORKS.—On December 16th Miss Thirza Green was appointed one of the Matrons of this asylum. She was trained at the South Yorkshire Asylum, where she afterwards had charge of the hospital ward for nine years. She holds a certificate from the Medico-Physiological Society.

DERBYSHIRE HOSPITAL FOR WOMEN.—Nurse Ada Jennings, on the completion of three years' training at Huddersfield General Infirmary, has been appointed to the post of Charge Nurse at the Derbyshire Hospital for Women.

GAINSBOROUGH UNION WORKHOUSE.—The newly-elected Head Nurse of this workhouse is Miss Edith Constance Joiner, who was trained at St. Olave's Infirmary, Rotherhithe.

### Notes and Queries.

*Training in Light Mental and other Nursing.*

(99) Can you tell me where a lady over thirty, who wishes to have a few months' training in nursing so that she could undertake slight mental or other cases, should apply? She would prefer being in London, and to be free of expense if possible.—*Inquirer.*

None of the London hospitals will receive probationers for a few months without a fee. But by looking through the lists of advertisements in "Mirror" for probationers, "Inquirer" might find some one to take her on reciprocal terms if she would train for one year. We do not recommend a short training, as it is always much more satisfactory to learn to do one thing well. In nursing, too, the second and third year nurses are paid, consequently it is well worth giving the extra time.

*Two Years or Three Years?*

(100) I was trained for two years at Lambeth Infirmary, and remained there two years longer as staff nurse, and I have recently been rejected as ineligible for the post of charge nurse under the Metropolitan Asylums Board because I have not a three years' certificate. What would you advise me to do? Enter for a three years' course at a London general hospital?—*Alecia.*

No doubt the advertisement was for a nurse holding a certificate of having been trained for three years at a training school answering to certain requirements, and if such a certificate could not be produced of course the applicant would be refused. When people enter as probationers at hospitals they should make strict inquiry as to the sort of certificates they will receive on leaving. There is a certain demand for nurses holding a one year certificate, but the tendency is to require a three years' certificate for all higher posts. It is, therefore, very desirable on entering a training school to have some assurance that if the probationer continues in the service of that hospital for three years she shall be able to continue her training during that time and receive a full certificate at the end of it. It is possible "Alecia," having been for four years at work in a training school, is already entitled to such a certificate. Inquiry should be made as to this of the matron of the Lambeth Infirmary. But when a nurse with a three years' certificate is advertised for, it would be unfair for one with a two years' certificate to be appointed over the heads of others who have complied with the terms of the advertisement.

*Ill-ventilated Closets.*

(101) What is the best disinfectant for use in ill-ventilated closets?—*Enquirer.*

There is no disinfectant that will take the place of ventilation. If, however, the ventilation is defective and cannot be improved, great care in keeping the pan clean by the use of soda is even more necessary than usual, and some permanganate solution may be poured down after use. If smell persists it is probable that the closet itself is defective, in which case it ought to be at once repaired. Pending this, however, the best plan is the free use of carbolic (1 to 40, made with the cheap No. 5 acid). But a large quantity must be poured quickly down twice a day, and the plumber is the cheaper expedient in the end.

*Homes for Private Nurses.*

(102) Will you kindly tell me if there are homes for private nurses in Rome or Florence?—*Nurse Hilda.*

Will make inquiries for you. Will any of our readers inform us from personal experience?

